# Supplementary material for: Chromothripsis during telomere crisis is independent of NHEJ, and consistent with a replicative origin
Source: Genome Res. 2019 May;29(5):737–49. doi: 10.1101/gr.240705.118 (PMC6499312; doi:10.1101/gr.240705.118)
Supplement: Supplemental Material [file supp_gr.240705.118_Supplemental_file_1.zip › contigs/annotated_contigs/DB91/contig.2.DB91_length_854_mean_cov_9.01053864169.docx]

**DB91_length_854_mean_cov_9.01053864169**

```
TGTGTTGCAAACCATAAGAGGATGTGCAAAAACAGTAGCTCCAAAGTGGAAAACAAAAGTAAAAATAATTCACATATTTTTTGCAGAAT
 >chr7:115552795-115553107 - E=9e-176 p=0e+00
TCTGATTCTCTTTCTTCTAACTTTCTTTCTTCCCCAGTGATTTGCTCTCAAAAGAGAAACATGAAAGTCAACAGTAAGCATATAAATAT

TAATAATTAAGACAGATGTCTGCAACTCACCTAAAATAAGAATGAAATTCTACTTGCAGAGTTCTTTTCTTTCACACACATGTGTGTGT

GTCTGTGTGTGTGTGTGAATGAAGCCCTCAAATATGGCACAATGT|TTAATAGG|TTAATTTCCTAACCTAAAACAAGCGCAAGAGATA
                                                      >chr7:115579371-115579905 + E=3e-
AAAATCTAAATGAAGCATCATAGTTATAAGTGCTTATAATAAATGCTAACAGTGCAACCAAAGATCATAGGACACTCTAAGTGTTTATA
288
GAAATAGAACCTTACGTTCCTCTCAGAACTGAAAATAATAAGAAATAGTTTGTTGTAATAATTATTTTGTCAATTGGCAATTCAATTTT

CAATATTTTATCCATTTGGAGAAAAACCAAATCAACAACTAATTGATTTCTGTCCAATACTGATGAAAAGTTTTAAGTACCTGAAAACT

GTCCTTGCAGGGGGACATTTTATATTTTATTTATTAAGATTGATGGTCGGAAATCTCTTCTTCATAAGAGAAGAGAATTGGGAATGTGA

TTCTGATGCAGTCAGAAGCATGAAGAAAACAAAAGAACATATACTACCTGAATGATGTGCATATGTGTATGTGTATGTTTGTGTGAAGG

GATACAATTCTGGTCCATATTCCTGAGGAAAATATTCAATATGTTCCTTTTCCTG
```
